# Variable Vulnerability: Genotype Determines Timing of PON1 Capability to Detoxify Pesticides

**Published:** 2009-10

**Authors:** Kris S. Freeman

**Affiliations:** **Kris S. Freeman** has written for *Encarta* encyclopedia, NIH, ABCNews.com, and the National Park Service. Her research on the credibility of online health information appeared in the June 2009 *IEEE Transactions on Professional Communication.*

Infants are extremely vulnerable to certain pesticide exposures because they are still developing the ability to produce the enzyme para‐oxonase‐1 (PON1), which detoxifies certain organophosphate pesticides such as chlorpyrifos and diazinon. A study published in 2003 indicated that children may reach near‐adult levels of PON1 activity by age 2 years. But a new larger‐scale study of participants in the Center for Health Assessment in Mothers and Children of Salinas cohort has found that many children are still ramping up PON1 levels until at least age 7 and that PON1 activity can vary dramatically among children of the same age **[*EHP*** **117:1632–1638; Huen et al.]**.

Organophosphates have been largely banned from home use but are still widely used in agriculture. These pesticides target the nervous systems of insects and also affect the human nervous system. Prior work by researchers involved in the current study revealed associations between prenatal exposure to organophosphate pesticides and increased reports of developmental delays and disorders in children.

Genetic variation in the *PON1* gene affects the type and quantity of the enzyme produced. A single‐nucleotide polymorphism at position 192 of the gene’s coding region changes the enzyme’s configuration, affecting its overall and pesticide‐specific efficiency; the R allele (form) of the PON1*_192_* polymorphism is more efficient than the Q allele at detoxifying organophosphate metabolites. A single‐nucleotide polymorphism at position − 108 of the gene’s promoter region affects the amount of PON1 produced, such that persons with the C allele have higher levels of PON1 activity than persons with the T allele.

In the current study, researchers measured PON1 activity from birth to age 7 years in 458 Mexican‐American children living in a heavily agricultural area. Blood samples were taken at up to five time points (birth, 1 year, 2 years, 5 years, and 7 years) and analyzed using three assays that measured PON1 quantity and efficiency. Blood samples were also genotyped for *PON1* variations. Researchers obtained a total of 1,143 samples, with 108 children providing samples at four or more time points.

Enzyme activity increased with age in all the children. However, compared with other children, PON1 activity was higher at birth and increased more quickly with age in children with the RR *PON1_192_* genotype or the CC *PON1_−108_* genotype (found in 24% and 28% of the children, respectively), such that in some cases average levels of activity against some pesticide metabolites were lower in children at age 7 than they were in other children at age 2. Children with both the RR and CC genotypes had the highest PON1 activity levels, and children with both the QQ and TT genotypes had the lowest levels.

These results suggest that some children may be vulnerable to the effects of organophosphate pesticides for longer than previously thought. The authors therefore recommend that policy makers consider this new information to ensure standards for pesticide exposures adequately protect young children.

